# Trends in Autism Research in the Field of Education in Web of Science: A Bibliometric Study

**DOI:** 10.3390/brainsci10121018

**Published:** 2020-12-21

**Authors:** Noemí Carmona-Serrano, Jesús López-Belmonte, Juan-Antonio López-Núñez, Antonio-José Moreno-Guerrero

**Affiliations:** 1 Ceuta Autism Association, University of Granada, 51001 Ceuta, Spain; nhoe@correo.ugr.es; 2 Department of Didactics and School Organization, University of Granada, 51001 Ceuta, Spain; ajmoreno@ugr.es; 3 Department of Didactics and School Organization, University of Granada, 18071 Granada, Spain; juanlope@ugr.es

**Keywords:** autism, bibliometric analysis, scientific mapping, SciMAT, Web of Science

## Abstract

Autism spectrum disorder (ASD) is conceived as a neurodevelopmental disorder. The scientific literature welcomes studies that reflect the possible singularities that people with ASD may present both in their daily lives and at an educational level. The main objective of this study is to analyze the scientific production on the term autism in Web of Science, focused on the educational field, in order to identify the research trends in this field of study. The intention is to offer researchers who study autism in the educational field some clear research directions. A bibliometric-type methodology was developed using the scientific mapping technique. For this purpose, a performance analysis and a co-word analysis were carried out. Work was conducted with an analysis unit of 5512 documents. The results show that the volume of production has been irregular from the beginning to the present. The collection of documents on the subject began to be relevant, in terms of the volume of production, from 2007, and this has persisted to the present. It is concluded that there are two lines of research. The first is the line focused on mothers of children with ASD and the second is the line of research focused on young people with ASD. In addition, since 2012, new lines of research have been generated, focused on the diagnosis and inclusion of these students in educational centers.

## 1. Introduction

Autism spectrum disorder (ASD), according to the Diagnostic and Statistical Manual of Mental Disorders in its fifth edition (DSM-V), is considered a multifactorial set of neurodevelopmental disorders [1]. It is characterized by a disorder in social [2] and communication skills and by the appearance of repetitive and stereotyped behaviors [3]. Additionally, people with ASD can display deficiencies in executive functioning, sensory–perceptual behavior, and attention, and they can also present symptoms of depression [4], challenging behaviors, restricted interests [5], and problems with emotional control [6] at higher levels than other people with typical development [7]. Another of the more superficial and concurrent symptoms that they can present is anxiety, which is currently being investigated [8]. All of this can increase exponentially if the person has a low cognitive ability [9].

All of these features can coexist with intellectual disability and impaired sensory processing [10]. Along these lines, people with ASD can present abnormal tactile behavior, despite being an important factor in all human relationships [11]. Likewise, experts postulate that the symptomatology focuses on the behavioral level [12].

People with ASD may exhibit an atypical response to the sounds they perceive in their environment, as they process sound stimuli differently from other people [13]. Similarly, people with ASD may face motor difficulties throughout their lives, which can have an impact on their quality of life and autonomy [14]. Moreover, people with ASD may display a deficiency in the visual processing of other people’s faces. This occurs due to a reported hypoactivation in the fusiform area of the face [15]. Similarly, people with ASD may demonstrate an impairment in planning their actions or tasks [16], as well as alterations in the structure and use of language [17].

Some children with ASD reveal writing skills in various ways, generally at a lower level than their peers [18]. On the contrary, they can process colors with a higher level of precision [19], and display better music processing skills than typically developing individuals [20].

At the gender level, science has revealed that women may exhibit less restricted and repetitive behaviors and attitudes than men. This assertion leads to alterations in brain networks based on gender, specifically those related to social integration and corticostriatal networks [21].

At the clinical level, the diagnosis of this pathology focuses on the phenotype. There is no reliable laboratory test available to assist healthcare personnel [22]. Furthermore, there is no pharmacological treatment for ASD, so its intervention is approached from a therapeutic perspective [23]. Approximately 1.5% of the world’s population suffers from this disorder [24].

### 1.1. ASD in the Field of Education

With regards to educational matters, students with ASD may have educational needs that other students do not. These can be caused by alterations or delays in the development of abilities that range from the fulfillment of instructions or rules to paying attention to the explanations of the teacher [25]. Additionally, these can include communication with other students and educational agents, which is a challenge for these types of people. Therefore, interventions that focus on interpersonal communication can lead to improvements in the social component of students with ASD [26].

An increasing number of students with ASD are attending regular schools [27]. Furthermore, there has been an increase in the number of students diagnosed with ASD enrolled in university [28]. Regarding the academic performance of people with ASD, the literature shows that there is a prevalence of a low performance or learning difficulty, but a gap is found in scientific studies focused on the high performance of people with ASD [29].

Early interventions may be a relevant factor for reducing the symptoms of people with ASD [30], as well as for improving and enhancing new skills [31,32]. These interventions should be based on observational learning, which has acquired a relevant value in people with ASD [33]. In this sense, the intervention should be aimed at acquiring skills to effectively carry out daily activities [34]. These activities can be carried out through theatrical practices that simulate reality, which have been demonstrated to exert great benefits, especially in terms of attention, empathy, collaboration, and anxiety reduction [35]. Science has shown to be emerging as a very important field of knowledge for people with ASD. This is due to the help it provides for understanding the world around them [36].

Considering this, the emotional regulation of people with ASD can be an important therapy for regulating behavioral problems and promoting sensory processing, with a favorable impact on social competence [4]. Along these lines, actions are being carried out focused on peer tutoring, that is, between students. This aims to improve the most deficient indicators and satisfy the needs of the students [37]. The lack of training and direct intervention by those around them or the professionals in their charge can lead to an increase in the social isolation of people with ASD [38]. Therefore, evidence-based, effective, and specialized practices and interventions are advocated for achieving inclusive education [39] and enabling students to achieve both social and academic goals [40]. In this sense, education professionals play a key role in the training of students with special educational needs [41]. This requires specialized training to meet all of the needs of such students [42]. Anything that is achieved in schools can have a positive impact on their future, as well as ensuring their autonomy and employability [43].

At the family level, interventions performed by those closest to students with ASD can be of great benefit [44]. Additionally, family empowerment can help relieve the stress and depression sometimes experienced by those closest to those with ASD. This is so important that it can reduce behavioral problems in children, as well as improve the mental health of the family as a whole [32]. Likewise, fluid communication between the educational institution and the family itself can lead to a reduction of stress and trust in parents, which facilitates the entire daily process [45]. Therefore, intervention and collaboration with families is essential. Parental programs are being developed that contribute to the reduction of anxiety for both students with ASD and their families [46]. Similarly, another of the actions that are being carried out to promote these links is the preparation of reports by the family to offer information to educational institutions. In this way, a more adequate performance is achieved by the professionals, thanks to the availability of complete and precise information on the singularities of the students [47].

The technology applied to people with ASD has been showing its potential. The application of new technological advances may lead to an increase in aspects such as liability [48]. The software developed is used to achieve autonomy and independence of the individual. However, the effectiveness of its design and interface continues to be studied today, with the aim of achieving greater suitability for people with ASD [49]. The development of innovative practices such as the use of augmented and virtual reality, as well as robotics, has led to improvements in various indicators, such as social skills, narrative, participation, communication, assimilation, and the retention of information [50,51,52,53]. However, on the regulatory side of emotions, technology has yet to show its best results [54].

Mobile technology is emerging as an effective resource for people with ASD in terms of helping them to develop socially and facilitate their communication needs, thus promoting inclusion in both the classroom and society itself [55]. In addition, new technological advances can allow them to be physically active and express their ideas and thoughts, without causing stress or anxiety [56]. Among the most outstanding advances are the training videos where each of the actions that the student has to carry out are explained in a guided manner [57,58]. A number of web pages that contain structured information that promotes learning, controlled by families and professionals, have also emerged [59]. In addition, the number of mobile applications aimed at training people with educational needs has increased, complementing the training received in educational centers [60]. In short, the possibilities of carrying out machine-assisted training have increased [61].

Despite the proliferation of digital resources, they are not being widely applied to the ASD community, and the adoption and application of technology by professionals who work with people with ASD is a challenge [62].

Regarding the teaching and learning methodology, today, gamified environments are on the rise; that is, a game-based learning space is generated, with rules, challenges, and tests to be performed collaboratively among students, which benefits social skills work [63]. Gamification promotes connection with a story in which students are involved, which fosters motivation, attention, and interest in tasks [64]. Furthermore, in this methodological line are serious games. These are games whose purpose is learning, rather than a playful activity. They encourage interactions between various agents, in different situations and contexts [65].

Regarding the means of detection, the most widely used instruments for the diagnosis and evaluation of people with ASD are based on questionnaires answered by professionals or caregivers. In this sense, observation acquires an important value [66].

### 1.2. Justification and Objectives

The literature presented has served to reveal the main findings on the state of the question by way of synthesis. In addition to establishing a definition of ASD, an overview of this disorder has been presented to reflect the needs that they may present, their educational situation, certain intervention guidelines, technological inclusion, family support, and the instruments used for the assessment of people with ASD.

However, there is a need to study ASD from a deeper perspective of the literature. In this work, the concept of “autism” provided in the documents registered in the Web of Science (WoS) database is analyzed from a bibliometric perspective [67]. The novelty of this study on the literature is the presentation of the concept analyzed by means of scientific mapping and through a dynamic and structural evolution of the state of the question to the scientific community. As a model, the research follows the analytical process carried out by recent studies extracted from the Journal Citation Reports (JCR) that have used this same documentary analysis technique [68].

In particular, the purpose of this study is to analyze the concept of ASD in the literature collected in WoS concerning the field of education. In the absence of similar works to the one presented in this study, we decided to approach this investigation with an exploratory nuance, from a generic perspective of the concept of education. The purpose of this study was to reduce the gap found in science and offer new findings to other researchers, with the intention of establishing bibliometric bases on the analyzed construct [69]. Therefore, this research pursues the following specific objectives:

- To know the performance of scientific production indexed in WoS on “autism” in the field of education
- To determine the scientific evolution of “autism” in the field of education in WoS
- To define the most decisive issues on “autism” in the field of education in WoS
- To find out the most frequently published authors on “autism” in the field of education in Wos

## 2. Materials and Methods

### 2.1. Research Design

Bibliometry was the methodology that supported this study, in order to achieve the proposed objectives. Bibliometry applies the potential of scientometry to carry out search, registration, analysis, and prediction actions in the scientific literature [70,71]. These actions were carried out following the guidance of experts in documentary analysis [72].

Specifically, this research focused on an analysis of co-words [73], as well as certain bibliometric indicators recommended by experts, such as the h, g, hg, and q^2^ indices [74]. This analysis gave rise to the realization of maps with nodes that could determine the performance and location of terminological subdomains [75] related to autism. In addition, these actions facilitated the study of conceptual thematic development in WoS [76].

### 2.2. Procedure and Data Analysis

The analytical procedure of this research was carried out at various points in time [77]: (1) Selection of the database under study. WoS was chosen because it is a database that covers the field of social sciences, including the field of education; (2) selection of the keyword (autism); (3) elaboration of the search equation (“autism” [TITLE] in the categories of “Education Educational Research”, “Education Scientific Disciplines”, “Education Special”, and “Psychology Educational”). We selected these categories because they are the ones that WoS uses to focus studies in the educational field; and (4) carrying out the search by grouping the metadata title, abstract, and keywords of the publications indexed in WoS. These actions resulted in a total of 6021 publications.

The results obtained allowed us to know the year in which autism research began to be published; in this case, it was 1963. Therefore, 1963–2019 was configured as the global study interval. Two main exclusion criteria were established in this study: (a) Documents published in 2020 (for not having finished the year; *n* = 396) and (b) repeated or poorly indexed documents in WoS (*n* = 113). These 113 documents were excluded because they did not focus on education. They were articles focusing on purely medical aspects. The application of these criteria produced a final analysis unit of 5512 documents. To show the results of the scientific performance and production, the following inclusion criteria were established: (a) Year of publication (all production except 2020); (b) language (x ≥ 10); (c) publication area (x ≥ 1000); (d) type of documents (x ≥ 200); (e) organizations (x ≥ 150); (f) authors (x ≥ 30); (g) sources of origin (x ≥ 200); (h) countries (x ≥ 200); and (i) the four most cited documents (x ≥ 430).

**Figure 1:**
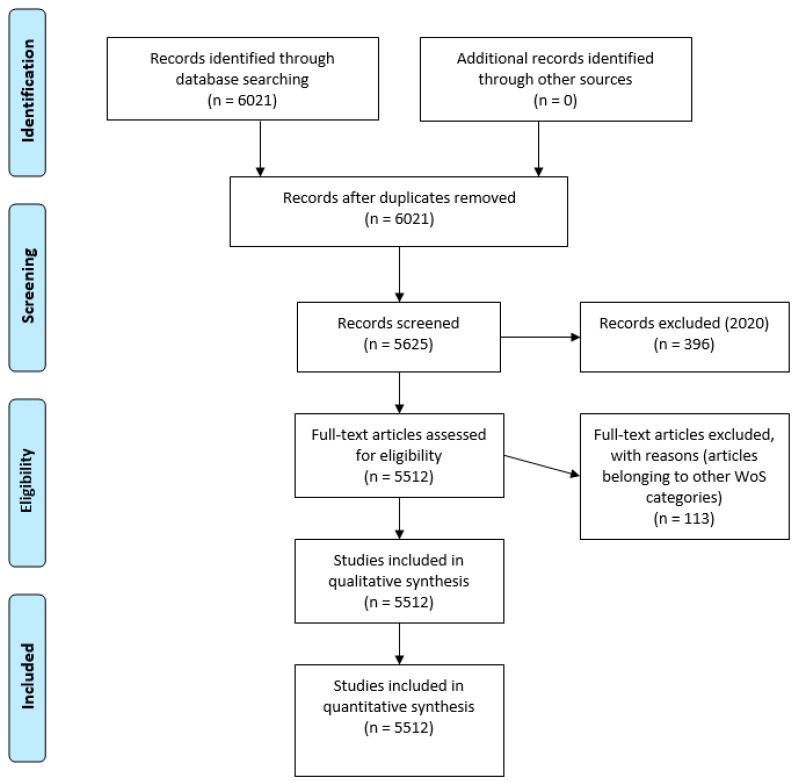
Flowchart according to the PRISMA Declaration.

The entire procedure is reflected in the following flow diagram based on the protocols of the PRISMA-P matrix (Figure 1).

Different programs were used to analyze the reported literature [78]. Analyze Results and Creation Citation Report (applications integrated in the WoS platform) were employed to extract and study the data related to the year, authorship, country, type of document, institution, language, medium, and most cited documents. On the other hand, the Science Mapping Analysis Tool (SciMAT), (Granada, Spain) was used to analyze the structural and dynamic development of the retrieved publications from a longitudinal perspective [79]. Throughout the analysis, the methodological and analytical guidelines of experts in SciMAT [80,81] were followed. With this last software, the following processes were carried out to conduct the analysis of co-words:

- Recognition: this process encompassed several actions: (1) the keywords of the reported WoS documents were analyzed (*n* = 9088); (2) co-occurrence node maps were designed; (3) a standardized network of co-words was created; (4) the keywords with the greatest significance were located (*n* = 8749); and (5) the most determining themes and constructs were established with a clustering algorithm
- Reproduction: various thematic networks and strategic diagrams made up of four regions were designed (upper right = motor and relevant themes; upper left = deeply rooted and isolated themes; lower left = disappearing or projected themes; and lower right = issues of poor development and transverse). All of these followed the principles of centrality and density
- Determination: Various temporary periods of literary study were configured. These were employed to analyze the development of nodes in different time intervals. Six periods were established (P_1_ = 1963–2008; P_2_ = 2009–2011; P_3_ = 2012–2013; P_4_ = 2014–2015; P_5_ = 2016–2017; and P_6_ = 2018–2019). The criteria for configuring the periods focused on originating intervals that had a similar number of publications. However, for authorship, only one period was configured, which occupies the entire analytical range (PX = 1963–2019). Likewise, to determine the strength of the association between intervals, the volume of keywords in common was taken as a reference
- Performance: This process involved the application of certain production indicators, connected to their corresponding inclusion criteria (Table 1)

## 3. Results

### 3.1. Scientific Performance and Production

**Figure 2:**
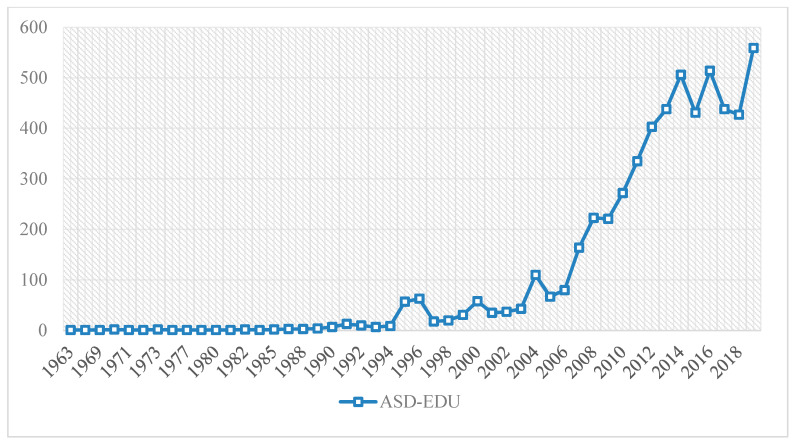
Evolution of scientific production.

The total scientific production on autism in the educational field (ASD-EDU) amounts to 5526 documents. These manuscripts have not evolved in a similar way and do not have a similar scientific production, in terms of the number of documents. Three clearly distinct stages can be observed. The first stage dates back to 1963, when research on ASD-EDU was first collected in WoS, and lasts until 1994. Throughout this period, production did not exceed 15 manuscripts per year. The second stage can be seen from 1995 to 2006, when the volume of production rises, although it does not exceed one hundred scientific productions per year. Finally, in the third stage, from 2007 to the present day, the volume of production is rising steeply. This rise in production is constant and continuous from 2007 to 2014. From 2014, the volume is maintained regularly, with peaks rising and falling, until 2019. The year 2019 is considered to be the year in which the most scientific production has taken place on ASD-EDU (Figure 2).

The language for excellence in ASD-EDU productions is English, which stands out from the other languages (Table 2).

The knowledge area where ASD-EDU studies are most frequently collected is Special Education, closely followed by Rehabilitation (Table 3).

In relation to the type of document used by the researchers, the research articles stand out considerably. The other types of documents exhibit a considerably lower value (Table 4).

There are three institutions that can be considered relevant to this field of study: Louisiana State University System; University of California System; and University of North Carolina. All of them are located in the United States (Table 5).

Of all the authors who have carried out research on ASD-EDU, Matson, J.L. stands out in terms of volume of production, with a total of 179 manuscripts. This is quadruple the volume of production of the second most frequently published author (Table 6).

As in other areas, there is one source of production that stands out above the rest, namely, Research in Autism Spectrum Disorders. This source of origin has a two-times higher value than that in second place. It is also worth noting that there is a high volume of production in the other journals about ASD-EDU (Table 7).

In relation to the country with the most scientific production, the United States stands out, with a high volume of manuscripts compared to the rest of the countries (Table 8).

Of all the existing reference documents on ASD-EDU, four stand out, mainly due to their high volume of citations received. In this case, they can be considered a reference within the scientific community. The first of these manuscripts is that by [82], with 633 citations. The aim of this study was to explore the influence of age and verbal ability on the theory of mental task execution. This study suggests that children with autism require a higher verbal mental age to pass false belief tasks than other typically developing children. The study by [83], with 570 citations, aims to examine the stress correlates of caregivers of young people with ASD. The results revealed that parents and teachers disagreed about the nature and severity of the behavioral problems. Instead, they stated that the behavioral problems were related to stress. The research by [84], with 562 citations, was based on an intensive behavioral intervention in two study groups of schoolchildren. The group of students with ASD had less restrictive school placements and a higher IQ than the group of typically developing students. It was concluded that behavioral treatment can generate important benefits in schoolchildren with ASD. Finally, the research by Bellini and [85], with 436 citations, develops a meta-analysis of the effectiveness of video modeling and self-modeling interventions in students with ASD. It is determined that these inverters allow working on social communication skills, functional skills, and behavioral functioning in children with ASD. They also show that these procedures promote the acquisition of skills and maintain them over time (Table 9).

### 3.2. Structural and Thematic Development

**Figure 3:**
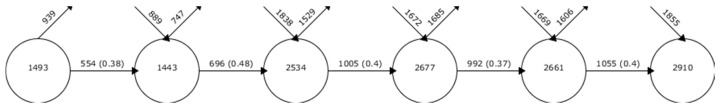
Continuity of keywords between contiguous intervals.

The beginning of the analysis started by investigating the evolution of keywords in the six established periods. This provided us with information on the level of coincidence of keywords between adjacent periods, and indicated the number of keywords that make up a period, which ones are absent in a specific period, and which ones are incorporated in a certain period. That is to say, the ascending arrows indicate the keywords that are eliminated with respect to the following period. The descending dates indicate the new keywords that are included in a period. The circles show the keywords of a period. The horizontal arrows show the words that match between periods. As shown in Figure 3, the level of coincidence between the periods is over 35%. This means that there is a high level of coincidence in the field of study of ASD-EDU. Therefore, it can be considered that there is a base established in this subject, with coincidences between researchers when it comes to establishing their lines of research.

The analysis of academic performance shows, from the themes resulting from the co-word analysis, the bibliometric values. The indicators shown are the h index, the g index, the hg index, and the q^2^ index. From the first period (1963–2008), the subject with the highest bibliometric value is “pervasive-developmental-disorders”, showing values that are much higher than the rest of the subjects. In the second period (2009–2011), the theme with the highest bibliometric value is “Young-children”, closely followed by “Asperger-syndrome”. In the third period (2012–2013), the theme with the highest bibliometric value is “children”, followed by “skills”. In the fourth period (2014–2015), the themes with the highest bibliometric value are “intervention” and “children”. In the fifth period (2016–2017), the subject matter with the highest bibliometric value is “children”. In the sixth and last period (2018–2019), the subject with the highest bibliometric value is “adolescents” (Table 10).

**Figure 4:**
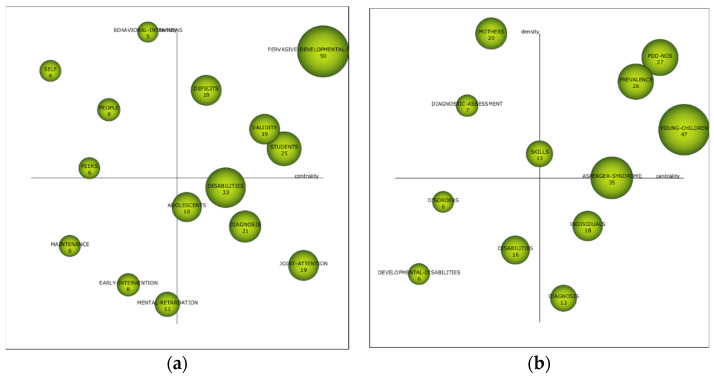

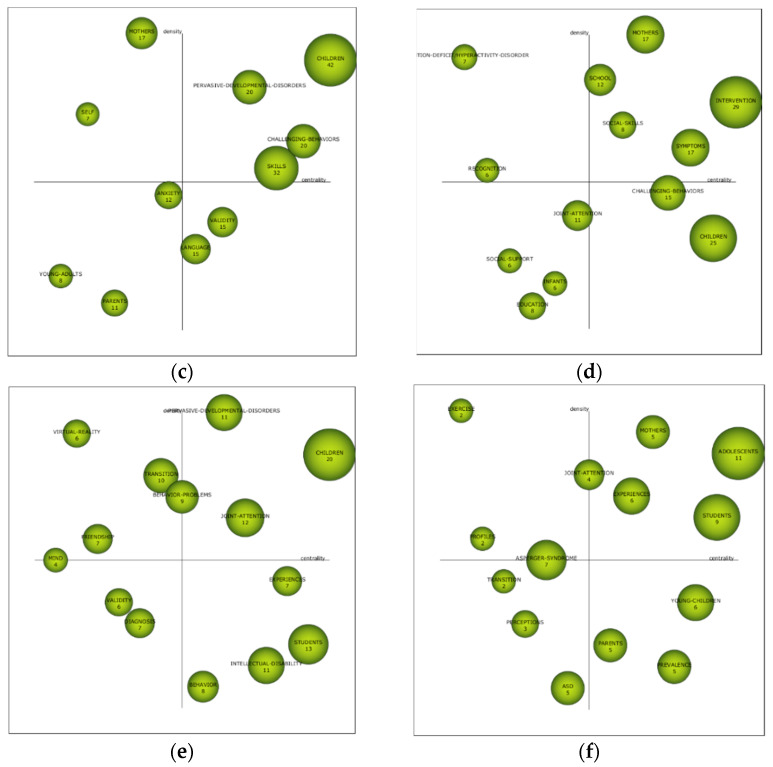
Strategic diagram constructed by ASD-EDU index h. Note: (**a**) interval 1963–2008; (**b**) interval 2009–2011; (**c**) interval 2012–2013; (**d**) interval 2014–2015; (**e**) interval 2016–2017; and (**f**) interval 2018–2019.

The analysis of strategic diagrams shows the importance, or relevance, of the themes obtained in the co-word analysis, according to the established periods. To do this, Callon’s index is taken into account. This diagram is divided into two levels: centrality, which shows the strength of the external connection with other themes or keywords, and density, which shows the strength of the internal connection of a network with other themes or keywords. The reference presented in each of the diagrams is the index h. Both diagrams and established periods have been generated, as shown in Figure 4. In addition, the keywords that relate to the driving issues are listed in Appendix A.

In the first period (1963–2008), the themes considered to be driving are “pervasive-developmental-disorders”, “deficits”, “validity”, and “students”. In this period, the most relevant studies focus on children’s disorder, the skills of young people with ASD, and the instruments with which to assess their various skills.

In the second period (2009–2011), the themes considered as driving forces are “PDD-NOS”, “prevalence”, and “young-children”. This period is notable for the identification of factors that cause ASD and the proper diagnosis of this type of child.

In the third period (2012–2013), the driving themes are “children”, “pervasive-developmental-disorders”, “challenging-behaviors”, and “skills”. In this period, children become relevant again, in relation to the skills and disabilities they present. In addition, attention is paid to challenging behavior.

In the fourth period (2014–2015), the driving themes are “mothers”, “school”, “intervention”, “social-skills”, and “symptoms”. In this period, the trend offered by the previous period changes, with the focus on mothers who have children with ASD, as well as the schools where they enroll. In this regard, research focuses on the harassment that students with ASD may experience in schools and on the possible problems that having a child with ASD may cause in the family.

In the fifth period (2016–2017), the driving themes are “pervasive-developmental-disorders”, “children”, and “joint-attention”. In this period, the trends of the first three periods are again taken up, with the focus of research on children. It is important to note that, during this period, the emphasis is on intervention with students with ASD.

In the sixth period (2018–2019), the driving themes are “mothers”, “experiences”, “adolescents”, and “students”. Furthermore, as this is the last period of analysis, the themes “transition” and “perceptions” must be taken into account, given that, due to their location in the diagram, they may be the new driving themes in the field of study of ASD-EDU. This last period focuses the relevance of ASD’s research on mothers, the educational experiences of students with ASD, young people, and students.

### 3.3. Thematic Evolution of Terms

The analysis of thematic evolution presents information related to the existing relationship between themes of contiguous periods. The relationship that can be established is of two types: conceptual, centered on a thematic relationship, and non-conceptual, centered on a relationship by means of keywords. The conceptual relationship is represented with a continuous line, while the non-conceptual relationship is related to a discontinuous line. The thickness of the line, either continuous or discontinuous, marks the number of themes or keywords for which there are coincidences. The greater the thickness, the greater the number of coincidences. The index used in this type of relationship is the Jaccard index.

**Figure 5:**
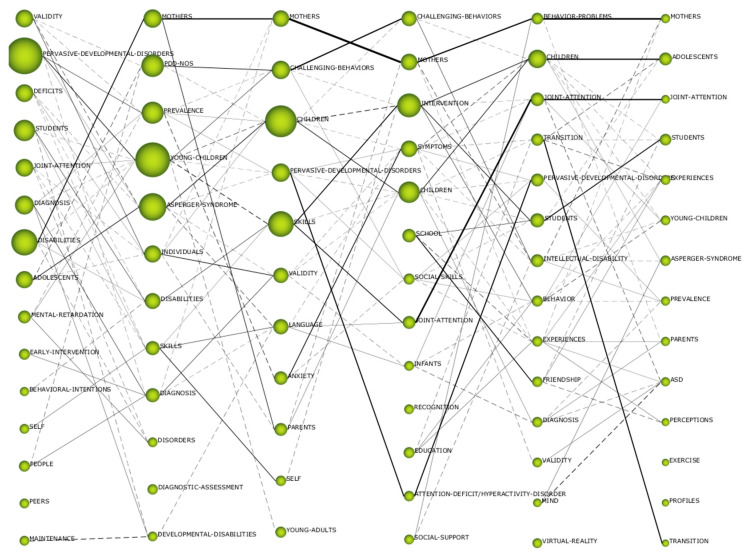
Thematic evolution by h index. *Note*: Continuous line (conceptual): thematic connection; discontinuous line (non-conceptual): keyword connection. Thematic columns, from left to right: column 1 (interval 1963–2008); column 2 (2009–2011); column 3 (interval 2012–2013); column 4 (interval 2014–2015); column 5 (interval 2016–2017); and column 6 (interval 2018–2019).

As can be seen in Figure 5, there is a conceptual gap in the field of ASD-EDU research. This means that there is no single theme that is repeated in all of the periods analyzed. It is true that there are themes that are repeated in various periods, but not in all of them. This does not mean that there are no time-based lines of research. In this case, two can be highlighted: “disabilities-mothers-mothers-behavior_problems-mothers” and “adolescents-Asperger_syndrome-children-children-adolescents”. In other words, in all periods, the lines of research focus on the children with ASD themselves, as well as on their mothers. In the third period (2012–2013), a new line of research emerged, focusing on “skills-joint_attention-joint_attention”; that is, on the care of people with ASD. Within this analysis, it can also be seen that there are more conceptual than non-conceptual relationships, which shows the relationships between the various lines of research.

### 3.4. Authors with the Highest Relevance Index

**Figure 6:**
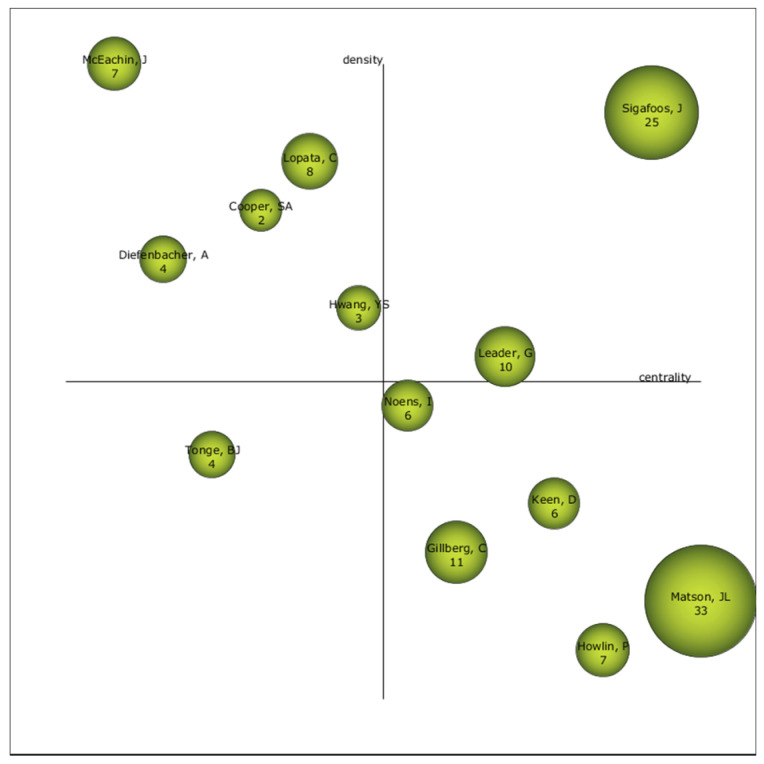
Strategic diagram of the author of all the productions by index h.

If we look at the relevance of the authors in the field of study of ASD-EDU, we see how Sigafoos, J, mainly, and Leader, G., can be considered the driving authors. This is due to their location in the diagram. Further, what is also noteworthy is the position of Tonge, B.J., which places him as one of the authors to be considered in the coming years, given that he may be the reference author in this field of study (Figure 6).

## 4. Discussion

ASD has been studied over the last six decades, as reflected in the results obtained. In the analysis of autism in the educational field, it can be seen how students can present problems in various capacities [2,3,4,5,9,10,11], as well as others that are an advantage [18,19] with respect to other people of typical development. In addition, special attention is paid to the use of technological resources to facilitate the satisfaction of these needs in order to achieve inclusion in learning spaces and society. Considering this, the objective of this research has focused on analyzing the scientific literature related to autism in the field of education available in WoS.

Scientific performance and production allow a profile to be established based on the general characteristics of ASD-EDU-related research. It can be indicated that the volume of production has been irregular from the beginning to the present. The collection of documents on the subject in WoS began to be relevant, in terms of production volume, in 2007, and persists to the present. Prior to this date, production was rather scarce. It is worth noting that the beginnings of studies, collected in WoS, date back to 1963.

The main means of presenting the results achieved in the various investigations by scientists are articles written in English. The fact that the results are presented through articles indicates that the research topic is well-established in the scientific community, having an adequate research and theoretical basis. The database where most studies on ASD-EDU are compiled is Education Special, which reveals that research on this subject focuses on disability itself. Furthermore, the main journal publishing this type of study is *Research in Autism Spectrum Disorders*. The high volume of production in the other journals presented in this manuscript is noteworthy. This marks the specialization of several journals in this field of study. In this case, they can be considered a reliable source of studies on ASD in education.

Among the most relevant institutions on the subject are Louisiana State University System, University of California System, and University of North Carolina. This shows the relevance of the United States in this type of research, as it is the country with the highest scientific production in the line of research analyzed.

The author with the highest volume of production is Matson, J.L. Although the most relevant authors are Sigafoos, J. and Leader, G., in the not-too-distant future, we must take into consideration the evolution of Tonge, B.J., who may be the reference author in the field of study of ASD-EDU. It is relevant that none of these authors are among the most cited in the educational community. Instead, the authors [82,83,84,85], whose research mainly focuses on various treatments applied to students with ASD, are the most cited.

From the analysis of keyword evolution, it is clear that there has been a solid and consistent research base over time in ASD-EDU’s field of study. The level of coincidence between the established periods is high, which makes it possible to see coincidences in the scientific community in charge of carrying out studies in this field of knowledge. In other words, the research topics are interrelated.

The analysis of academic performance shows that there are changes between the different periods analyzed, in terms of the subjects with the highest bibliometric values. However, of all these periods, one theme that is usually repeated can be observed, which is either a child or a young person; that is, the trend in educational research is to focus on young people with ASD. Then, depending on the period, the theme varies, with attention also being paid to skills and interventions.

The strategic diagram analysis provides information on the degree of importance of the different themes, in each of the established periods. In the first period (1963–2008), the most relevant themes are focused on students with ASD and on the specificities of the effects they generate in children. It also focuses on the instruments for assessing and analyzing the skills of children with ASD. The second period (2009–2011) evolves towards the importance of diagnosing and identifying children with ASD, as well as determining the specific skills that each of them presents. The third period (2012–2013) follows the trend of the two previous periods, where the most relevant research focuses on children and the diagnosis of their skills, although the focus of research is on the challenging behaviors of students with ASD. In the fourth period (2014–2015), there is a change in the trend of research relevance. In this case, the focus is on schools and families, especially on the problems that can be generated by having a student with ASD. In the fifth period (2016–2017), trends from the first periods are again being taken up, focusing research on the children themselves, although importance is beginning to be given to interventions with students who have ASD. The sixth and final period (2018–2019) shows a connection between the aspects analyzed in each of the previous periods, mainly focusing on mothers, young people, educational experiences, and students with ASD. In addition, the themes of “transition” and “perceptions” should be borne in mind, as they may be the driving forces in the coming years. It is important to note that there is no single theme that is a driving force in all of the periods analyzed. These driving forces vary from one period to another, although there are some, such as “students”, “mothers”, “children”, and “pervasive developmental disorders”, that are repeated, at least several times, in different periods.

The thematic evolution of ASD-EDU shows that the two main lines of research are focused on mothers and on children with ASD themselves, although a new line of research has emerged since 2012, which focuses on the intervention of young people with ASD. As far as can be seen from this analysis, there is a consistent and well-established trend in research on ASD-EDU, with strong links between the various study topics.

## 5. Conclusions

It can be concluded that the subject of study on ASD-EDU in WoS dates back to 1963, but it was not until 2007 that the volume of production and research in this field of study increased considerably. Throughout this period, two clearly differentiated but closely related lines of research stand out: Research on the mothers of children with ASD and on the young people with ASD themselves. Since 2012, intervention with this type of student has been added to these two established lines of research. In addition, in general, research on ASD-EDU has also focused on diagnosis and the inclusion of students with ASD in educational centers.

The prospective of this research is oriented towards offering the scientific community the research trends in this field of study, in order to show them the most relevant aspects. It also allows teachers who deal with this type of student a source of information, so that they can look for and investigate research with more impact.

The limitations of the study are focused on the purification of the database, which entails a considerable effort and work for researchers, given that they must analyze, in the volume of work presented, all studies in order to discard those that are not related to this line of research. Furthermore, the inclusion values established are for the consideration of the researchers themselves, so that the variation in them can generate changes in the results obtained in this study. As future lines of research, a co-word analysis on the family and ASD should be carried out, in order to deepen and analyze, in more detail, the type of studies that are developed in this specific field of research. Furthermore, we intend to compare these results with other databases, such as Scopus and PubMed. Additionally, a future line of research should be focused on analyzing, following the techniques of systematic reviews of the literature, the various existing educational approaches for ASD.

This study has certain educational implications, given that, during the analysis, various intervention techniques could be observed with students with ASD, as well as various tools for their diagnosis and inclusion in educational centers. This study also has theoretical implications, since it offers the scientific community information on the most relevant lines of research in this field of study, as well as indicating possible trends in research on ASD-EDU. In other words, this study allows teachers to see which journals deal with the term autism in education with the greatest volume of production, which authors are the most productive regarding ASD-EDU, which country has the highest production on ASD-EDU, and which document about ASD-EDU is the most cited, among other significant results. That is to say, the data represented allow the teacher to locate relevant and updated information, in order to improve their pedagogical actions. Moreover, they can permit researchers to analyze the most relevant and interesting lines of research developed by the scientific community.

## Figures and Tables

**Table 1:** Production indicators and inclusion criteria.

Configuration	Values
Analysis unit	Keywords authors, keywords Web of Science (WoS)
Frequency threshold	Keywords: P_1_ = (4), P_2_ = (4), P_3_ = (5), P_4_ = (5), P_5_ = (5), P_6_ = (5)
	Authors: P_X_ = (8)
Network type	Co-occurrence
Co-occurrence union value threshold	Keywords: P_1_ = (3), P_2_ = (3), P_3_ = (4), P_4_ = (4), P_5_ = (4), P_6_ = (4)
	Authors: P_X_ = (5)
Normalization measure	Equivalence index
Clustering algorithm	Maximum size: 9; Minimum size: 3
Evolutionary measure	Jaccard index
Overlapping measure	Inclusion rate

**Table 2:** Scientific language used.

Languages	*n*
English	5487
Spanish	58
Turkish	41
Portuguese	18

**Table 3:** Areas of knowledge.

Areas of Knowledge	*n*
Special Education	4307
Rehabilitation	3538
Psychiatry	1810
Developmental Psychology	1763

**Table 4:** Type of document.

Document Types	*n*
Article	4015
Meeting abstract	605
Proceeding paper	357
Review	288
Book review	237

**Table 5:** Institutions.

Denomination	*n*
Louisiana State University System	191
University of California System	172
University of North Carolina	167

**Table 6:** Most prolific authors.

Authors	*n*
Matson, J.L.	179
Sigafoos, J.	42
Lang, R.	38
Gillberg, C.	36
Hasting, R.P.	31
Howlin, P.	31

**Table 7:** Source of origin.

Denomination	*n*
*Research in Autism Spectrum Disorders*	1123
*Journal on Intellectual Disability Research*	612
*Research in Developmental Disabilities*	356
*Journal of Developmental and Physical Disabilities*	215
*Focus on Autism and Other Developmental Disabilities*	206

**Table 8:** Most prolific countries.

Countries	*n*
USA	2821
England	567
Australia	469
Canada	269

**Table 9:** Most cited articles.

References	Citations
[81]	633
[82]	570
[83]	562
[84]	436

**Table 10:** Thematic performance in autism in the educational field (ASD-EDU).

**Interval 1963–2008**						
**Denomination**	**Works**	**Index h**	**Index g**	**Index hg**	**Index q^2^**	**Citations**
Validity	26	19	25	21.79	32.03	1316
Pervasive-developmental-disorders	137	50	86	65.57	70.36	8001
Deficits	27	19	27	22.65	36.47	2293
Students	49	25	47	34.28	33.17	2291
Joint attention	29	19	28	23.07	35.41	1769
Diagnosis	34	21	34	26.72	32.4	1702
Disabilities	44	33	43	37.67	46.31	3804
Adolescents	27	18	26	21.63	24.74	1420
Mental-Retardation	14	11	14	12.41	27.95	1119
Early-intervention	9	8	9	8.49	16.97	644
Behavioral-intentions	5	5	5	5	13.42	193
Self	6	6	6	6	24.98	561
People	8	8	8	8	29.8	1429
Peers	7	6	7	6.48	13.42	200
Maintenance	6	6	6	6	14.7	216
**Interval 2009–2011**						
**Denomination**	**Works**	**Index h**	**Index g**	**Index hg**	**Index q^2^**	**Citations**
Mothers	27	20	27	23.24	35.78	1707
PPD-NOS	38	27	38	32.03	36.74	2301
Prevalence	38	26	38	31.43	43.86	2305
Young-children	169	47	76	59.77	64.68	7420
Asperger-Syndrome	66	35	60	45.83	50.2	3626
Individuals	23	18	23	20.35	25.811	970
Disabilities	23	16	23	19.18	22.27	764
Skills	18	13	18	15.3	25.5	842
Diagnosis	16	13	16	14.42	23.37	808
Disorders	7	6	7	6.48	22.45	676
Diagnostic-assessment	7	7	7	7	19.8	708
Developmental-disabilities	6	6	6	6	10.39	126
**Interval 2012–2013**						
**Denomination**	**Works**	**Index h**	**Index g**	**Index hg**	**Index q^2^**	**Citations**
Mothers	26	17	26	21.02	26.08	788
Challenging-behaviors	49	20	30	24.49	26.83	1048
Children	339	42	62	51.03	51.85	7821
Pervasive-developmental-disorders	47	20	34	26.08	28.64	1243
Skills	101	32	48	39.19	40.4	2841
Validity	33	15	23	18.57	19.36	576
Language	35	15	24	18.97	21.21	645
Anxiety	19	12	19	15.1	25.22	718
Parents	13	11	13	11.96	16.91	387
Self	8	7	8	7.48	8.77	134
Young-adults	8	8	8	8	15.23	313
**Interval 2014–2015**						
**Denomination**	**Works**	**Index h**	**Index g**	**Index hg**	**Index q^2^**	**Citations**
Challenging-behaviors	43	15	24	18.97	20.86	673
Mothers	48	17	25	20.62	20.62	797
Intervention	320	29	40	34.06	34.9	4142
Symptoms	51	17	26	21.02	20.2	865
Children	167	25	34	29.15	30.82	2194
School	27	12	19	15.1	16.97	411
Social-Skills	30	8	13	10.2	10.58	231
Joint-Attention	24	11	19	14.46	16.25	382
Infants	14	6	10	7.75	8.49	109
Recognition	11	6	10	7.75	8.49	118
Education	12	8	11	9.38	14.7	251
ADHD	11	7	10	8.37	9.9	142
Social-Support	8	6	8	6.93	14.07	170
**Interval 2016–2017**						
**Denomination**	**Works**	**Index h**	**Index g**	**Index hg**	**Index q^2^**	**Citations**
Behavior-problems	23	9	14	11.22	11.62	245
Children	392	20	27	23.24	24.08	2577
Joint-attention	64	12	17	14.28	15.1	438
Transition	35	10	15	12.25	13.78	302
Pervasive-developmental-disorders	27	11	16	13.27	14.46	311
Students	74	13	18	15.3	15.72	529
Intellectual-disability	36	11	14	12.41	12.85	313
Behavior	36	8	12	9.8	11.66	219
Experiences	32	7	14	9.9	10.58	253
Friendship	18	7	12	9.17	9.9	161
Diagnosis	16	7	11	8.77	9.54	133
Validity	12	6	8	6.93	7.35	75
Mind	9	4	8	5.66	8.94	91
Virtual-reality	10	6	9	7.35	6.93	95
**Interval 2018–2019**						
**Denomination**	**Works**	**Index h**	**Index g**	**Index hg**	**Index q^2^**	**Citations**
Mothers	56	5	6	5.48	5.92	136
Adolescents	286	11	15	12.85	13.27	784
Joint-attention	47	4	6	4.9	6.63	84
Students	117	9	11	9.95	9.995	267
Experiences	42	6	8	6.93	8.49	119
Young-children	81	6	9	7.35	9.49	168
Asperger-syndrome	36	7	12	9.17	9.9	193
Prevalence	33	5	8	6.32	6.71	105
Parents	38	5	5	5	5	84
ASD	31	5	6	5.48	5.48	82
Perceptions	13	3	4	3.46	3.46	21
Exercise	5	2	3	2.45	4.69	18
Profiles	8	2	4	2.83	4	17
Transition	9	2	3	2.45	3.74	18

## Appendix A

**Table A1:** Relationship between keywords and driving themes.

**Interval 1963–2008**	
**Driving Themes**	**Keywords**
Pervasive-developmental-disorders	Childhood-autism, epidemiology, infantile-autism, age, spectrum-disorders, autism, Asperger-syndrome, and young-children.
Deficits	Memory, play, normal-children, executive-function, mind, performance, individual, and symbolic-play.
Validity	Reliability, differential-diagnosis, issues, children, impairment, intellectual-disability, interview, and classification.
Students	Skills, inclusion, acquisition, behavior, instruction, outcomes, peer-interactions, and school.
**Interval 2009–2011**	
**Driving Themes**	**Keywords**
PDD-NOS	Differential-diagnosis, Matson-evaluation, profound-mental-retardation, adults, challenging-behavior, self-injurious-behavior, social-skills, and Asperger-syndrome.
Prevalence	Psychopathology, epidemiology, epilepsy, intellectual-disability, pervasive-developmental-disorders, psychiatric-disorders, rates, and population.
Young-children	Joint-attention, mental-retardation, biscuit, Autism-Spectrum-Disorder, behavior, intervention, and autism.
**Interval 2012–2013**	
**Driving Themes**	**Keywords**
Children	Asperger-syndrome, prevalence, individuals, adolescents, adults, Autism-Spectrum-Disorder, instruction, and autism.
Pervasive-developmental-disorders	Gender, childhood-autism, diagnostic-interview, spectrum-disorders, ADHD, and symptoms.
Challenging-behaviors	Young-children, aggression, biscuit, intellectual-disability, risk-factors, self-injurious-behavior, social-skills, and stereotype.
Skills	Technology, joint-attention, imitation, behavior, communication, disabilities, intervention, and students.
**Interval 2014–2015**	
**Driving Themes**	**Keywords**
Mothers	Mental-health, parenting-stress, syndrome-specificity, adjustment, behavior-problems, families, fathers, and stress.
School	Inclusion, bullying, loneliness, disorders, friendship, perceptions, perspectives, and victimization.
Intervention	Autism, young-children, individuals, autism-spectrum-disorders, behavior, skills, students, and video-modeling.
Social-skills	DSM-IV, Matson-evaluation, diagnostic-assessment, ASD, Depressive-behavioral-intervention, pervasive-developmental-disorder, social-stories, and play.
Symptoms	Anxiety, depression, reliability, abnormalities, adolescents, psychiatric-disorders, toddlers, and youth.
**Interval 2016–2017**	
**Driving Themes**	**Keywords**
Pervasive-developmental-disorders	Mental-retardation, population, psychopathology, ADHD, prevalence, symptoms, and mental-retardation.
Children	Autism, Asperger-syndrome, anxiety, adolescents, adults, Autism-spectrum-disorders, intervention, spectrum-disorders, and autism.
Joint-attention	Language, pre-school-children, language-development, communication, infants, toddlers, Young-children, and pretend-play.
**Interval 2018–2019**	
**Driving Themes**	**Keywords**
Mothers	Stress, mental-health, impact, behavior-problems, families, fathers, social-support, and quality-of-life.
Experiences	University, interpretative-phenomenological-analysis, Young-people, friendship, spectrum-disorders, NEEDS, education, and higher-education.
Adolescents	Individuals, anxiety, high-school, adults, autism-spectrum-disorder, children, young-adults, and youth.
Students	Inclusion, comprehension, elementary, disabilities, instruction, school, skills, and autism.
